# Investigating auranofin for the treatment of infected diabetic pressure ulcers in mice and dermal toxicity in pigs

**DOI:** 10.1038/s41598-021-90360-x

**Published:** 2021-05-25

**Authors:** Haroon Mohammad, Nader S. Abutaleb, Alexandra M. Dieterly, L. Tiffany Lyle, Mohamed N. Seleem

**Affiliations:** 1 Department of Comparative Pathobiology, College of Veterinary Medicine, Purdue University, 625 Harrison St., West Lafayette, IN 47907 USA; 2 Center for One Health Research, Department of Biomedical Sciences and Pathobiology, Virginia-Maryland College of Veterinary Medicine, Virginia Polytechnic Institute and State University, 1410 Prices Fork Rd, Blacksburg, VA 24061 USA; 3 Center for Comparative Translational Research, Purdue University, 625 Harrison St., West Lafayette, IN 47907 USA

**Keywords:** Bacterial infection, Drug discovery

## Abstract

Bacterial infection of pressure ulcers (PUs) are a notable source of hospitalization for individuals with diabetes. This study evaluated the safety profile and efficacy of auranofin to treat diabetic PUs infected with methicillin-resistant *Staphylococcus aureus* (MRSA). PUs were infected with MRSA in diabetic TALLYHO/JngJ mice and then treated with topical auranofin (2%), topical mupirocin (2%), or oral clindamycin (30 mg/kg) for four days. PUs were harvested post-treatment to enumerate bacterial burden and determine expression of cytokines/growth factors. Landrace cross pigs were exposed topically to auranofin (1%, 2%, and 3%) for 4–14 days and evaluated for signs of localized or systemic toxicity. Auranofin eradicated MRSA in PUs within four days (7.92-log_10_ reduction) in contrast to mupirocin (2.15-log_10_ reduction) and clindamycin (0.73-log_10_ reduction). Additionally, auranofin treatment resulted in decreased expression of pro-inflammatory cytokines and increased expression of biomarkers associated with re-epithelization of wounded tissue, confirmed with histopathologic analysis. No significant histopathologic lesions were present on porcine skin sites exposed to topical auranofin. Additionally, minimal accumulation of plasma gold and no systemic toxicity was observed in pigs exposed to topical auranofin. Auranofin appears to be a potent and safe topical agent to further investigate for treatment of mild-to-moderate MRSA-infected diabetic PUs.

## Introduction

Diabetes is a significant global public health challenge that affects approximately 9% of all adults (463 million people) and is associated with 4.2 million deaths each year^1,2^. Diabetes is a chronic disease that increases the risk for development of pressure ulcers in the lower extremities due to neuropathy and poor blood circulation^1,2^. A pressure ulcer results when the skin and underlying tissue are compressed, typically between a bone and an external surface, for an extended period, which leads to tissue damage or necrosis. Pressure ulcers are graded based upon severity and range from Stage 1, which are mild and typically present as erythema with intact skin, to Stage 4, which includes damage to the skin and underlying tissue including bone, muscle, and tendons^3^. In patients with diabetes, pressure ulcers frequently develop on the feet. Globally, 2% of adults will develop a diabetic foot ulcer (DFU) each year resulting in over 9.1 million DFUs annually^1,4,5^. Treatment of DFUs has been estimated to range from $9 to $13 billion annually in the U.S. and is linked to one-third of the total medical costs attributed to diabetes^5,6^.

Nearly half of all DFUs that present in a healthcare setting are infected with bacteria^4,7^. Bacterial infection of diabetic pressure ulcers presents a significant treatment challenge. Infections can contribute to impaired wound healing and can spread to underlying tissues resulting in osteomyelitis, systemic infection, increased risk of amputation, and death^8–10^. As a result, nearly 1% of individuals with diabetes will experience amputation of at least one lower limb^1^. Additionally, the cost to treat an infected DFU nearly doubles in comparison to treatment of uninfected pressure ulcers^11^. *Staphylococcus aureus* is the most frequently isolated pathogen from infected DFUs in Western nations^5,8^. The emergence of strains of methicillin-resistant *S. aureus* (MRSA) combined with *S. aureus*’ ability to form robust biofilms has contributed to treatment failure of infected DFUs^5,8,11^. Though antibiotics used to treat infected DFUs are capable of reducing the burden of bacteria in the ulcerated tissue, they do not enhance or promote early wound healing, including re-epithelization of the damaged tissue. Discovering agents capable of rapidly reducing bacterial burden in infected diabetic pressure ulcers that also reduce inflammation and enhance wound healing are needed.

Auranofin is an FDA-approved drug originally indicated for the treatment of rheumatoid arthritis in 1985. Since its discovery, researchers have attempted to reposition auranofin as a novel antibacterial, antifungal, antiparasitic, and antiviral agent^12–18^. This effort has proven successful as auranofin received orphan drug status for the treatment of amebiasis^19^. Auranofin has been shown to exhibit potent antibacterial and antibiofilm activity against MRSA in vitro and in vivo in murine cutaneous abscess and mesh-associated biofilm models^16,20^. We hypothesized that the potent antibacterial, antibiofilm, and anti-inflammatory activities of auranofin would render it a promising new candidate for the treatment of MRSA-infected pressure ulcers. The objectives of the present study were to evaluate auranofin in the treatment of MRSA-infected pressure ulcers in diabetic mice and to evaluate the safety profile of auranofin as a topical agent when applied to porcine skin. Addressing efficacy and safety in these animal models is an important pre-clinical step to repurpose auranofin for the treatment of mild-to-moderate MRSA-infected pressure ulcers in diabetic patients.

## Results

### Auranofin rapidly eradicates MRSA in infected pressure ulcers in diabetic mice

**Figure 1:**
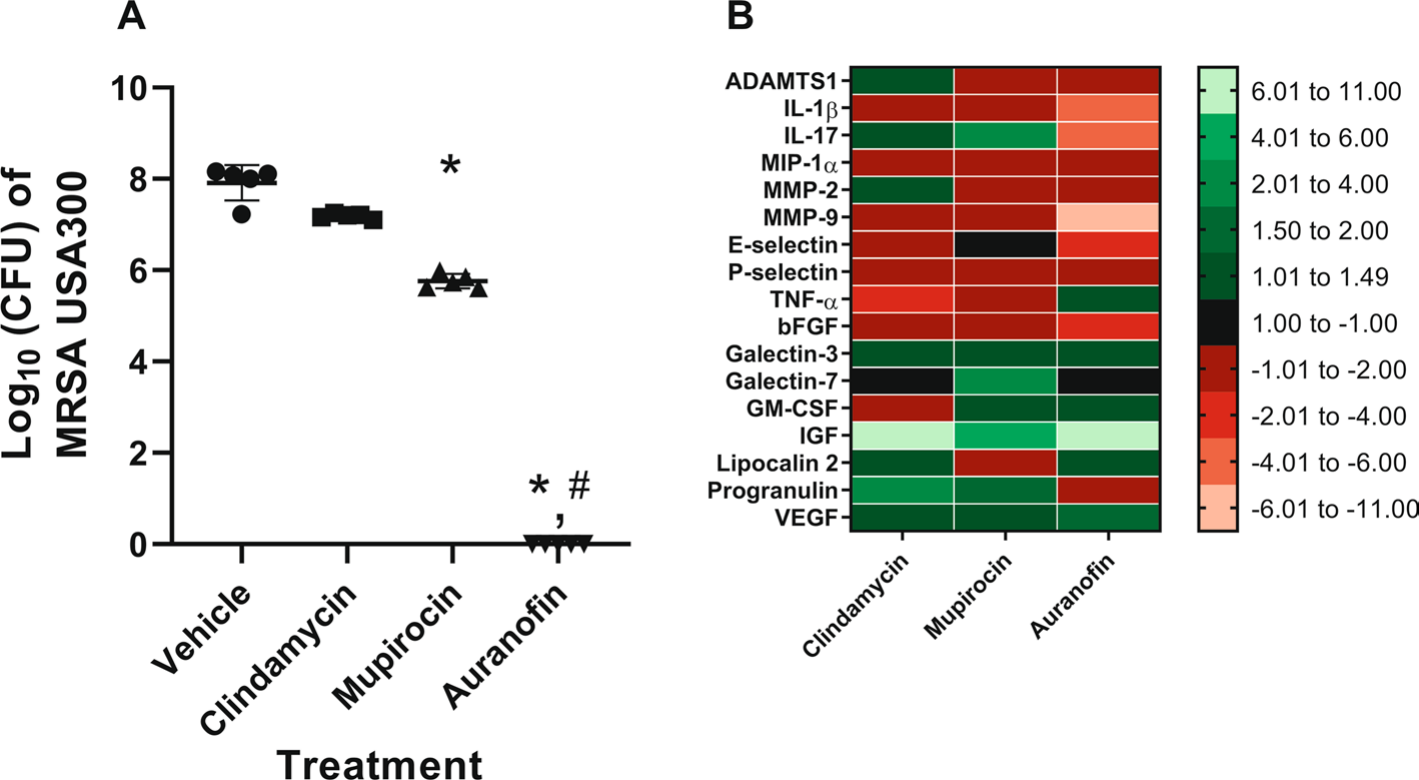
Bacterial burden and cytokines expression in pressure ulcers treated with auranofin and control antibiotics in diabetic mice. Male TALLYHO/JngJ mice (n = 5) were exposed to magnets to induce the formation of pressure ulcers that were subsequently infected with MRSA USA300. Infected pressure ulcers were treated with oral clindamycin (30 mg/kg, q.d.), topical mupirocin (2%), topical auranofin (2%), or vehicle alone (petroleum jelly) b.i.d. for four days. Twelve hours after the final dose, mice were euthanized, and the infected PUs were aseptically removed to determine bacterial burden and expression of cytokines related to inflammation and re-epithelization of wounded tissue. (**A**) MRSA USA300 colonies present in pressure ulcers after treatment with the vehicle alone, control antibiotics, or auranofin. Data are presented as log_10_ (total MRSA CFU per wound) for each mouse and were evaluated using the Kruskal–Wallis test with Dunn’s multiple comparisons test. An asterisk (*) indicates statistical difference for test agents relative to petroleum jelly (negative control, *P* < 0.05) while a pound sign (#) indicates statistical difference between mice treated with auranofin compared to clindamycin (*P* < 0.05). (**B**) Fold-change in expression of cytokines and growth factors in pressure ulcers treated with control antibiotics or auranofin (after four days) relative to PUs treated with the vehicle alone, as determined by the Quantibody Mouse Cytokine Array 4000 kit.

Male diabetic TALLYHO/JngJ mice exhibiting moderate MRSA-infected pressure ulcers were treated with oral clindamycin (30 mg/kg) q.d. or topically with auranofin (2%), mupirocin (2%), or vehicle alone b.i.d. for four days. The burden of MRSA USA300 in infected PUs was determined thereafter (Fig. 1A). Complete eradication of MRSA (7.92-log_10_ reduction) was observed in PUs treated with auranofin, which was superior to clindamycin (*P* = 0.0181). Mupirocin successfully reduced bacterial burden by 2.15-log_10_ while clindamycin reduced MRSA burden in infected PUs by 0.73-log_10_. Auranofin (*P* = 0.0002) and mupirocin (*P* = 0.0249) both generated a statistically significant reduction in MRSA burden in infected PUs relative to the vehicle alone.

### Evaluation of auranofin and control antibiotics on expression of markers related to inflammation and wound healing in infected diabetic pressure ulcers

Pressure ulcers infected with MRSA and treated with auranofin or control antibiotics were next evaluated for signs of wound healing (Fig. 1B). Expression levels for seventeen biomarkers related to inflammation and growth factors involved in tissue re-epithelization were evaluated and compared relative to PUs treated with the vehicle alone. Auranofin reduced expression of multiple pro-inflammatory cytokines including IL-1β (−5.00-fold change) and IL-17 (−4.03-fold change). Additionally, treatment with auranofin resulted in decreased expression of matrix metalloproteinases (MMP) including MMP-2 (−1.67-fold change) and MMP-9 (−6.28-fold change). There was minimal difference observed in the expression of tumor necrosis factor-alpha (TNF-α) (1.39-fold difference) for PUs treated with auranofin compared to the vehicle alone. Furthermore, auranofin treatment resulted in increased expression of factors that play an important role in re-epithelization of wounded tissue including granulocyte–macrophage colony-stimulating factor (GM-CSF) (1.39-fold change), insulin-like growth factor (IGF) (10.53-fold change), lipocalin 2 (1.18-fold change), and vascular endothelial growth factor (VEGF) (1.78-fold change).

Treatment of PUs with mupirocin resulted in a slight decrease in expression of IL-1β (−1.32-fold change), MMP-2 (−1.28-fold change), MMP-9 (−1.16-fold change), and TNF-α (−1.87-fold change). Additionally, increased expression of GM-CSF (1.49-fold change), IGF (4.99-fold change), and VEGF (1.35-fold change) was observed in PUs treated with topical mupirocin. Similar to mupirocin, mice treated with oral clindamycin exhibited a decrease in expression of IL-1β (−1.32-fold change), MMP-2 (−1.37-fold change), and TNF-α (−2.80-fold change) in infected pressure ulcers. Expression of IGF (6.47-fold change), lipocalin 2 (1.23-fold change), and VEGF (1.42-fold change) were increased in infected PUs of clindamycin-treated mice while GM-CSF expression (−1.06) was slightly decreased. PUs from mice treated with mupirocin (2.25-fold change) or clindamycin (1.35-fold change) resulted in increased expression of IL-17.

### Histological evaluation of MRSA-infected pressure ulcers in diabetic mice treated with auranofin and control antibiotics

**Figure 2:**
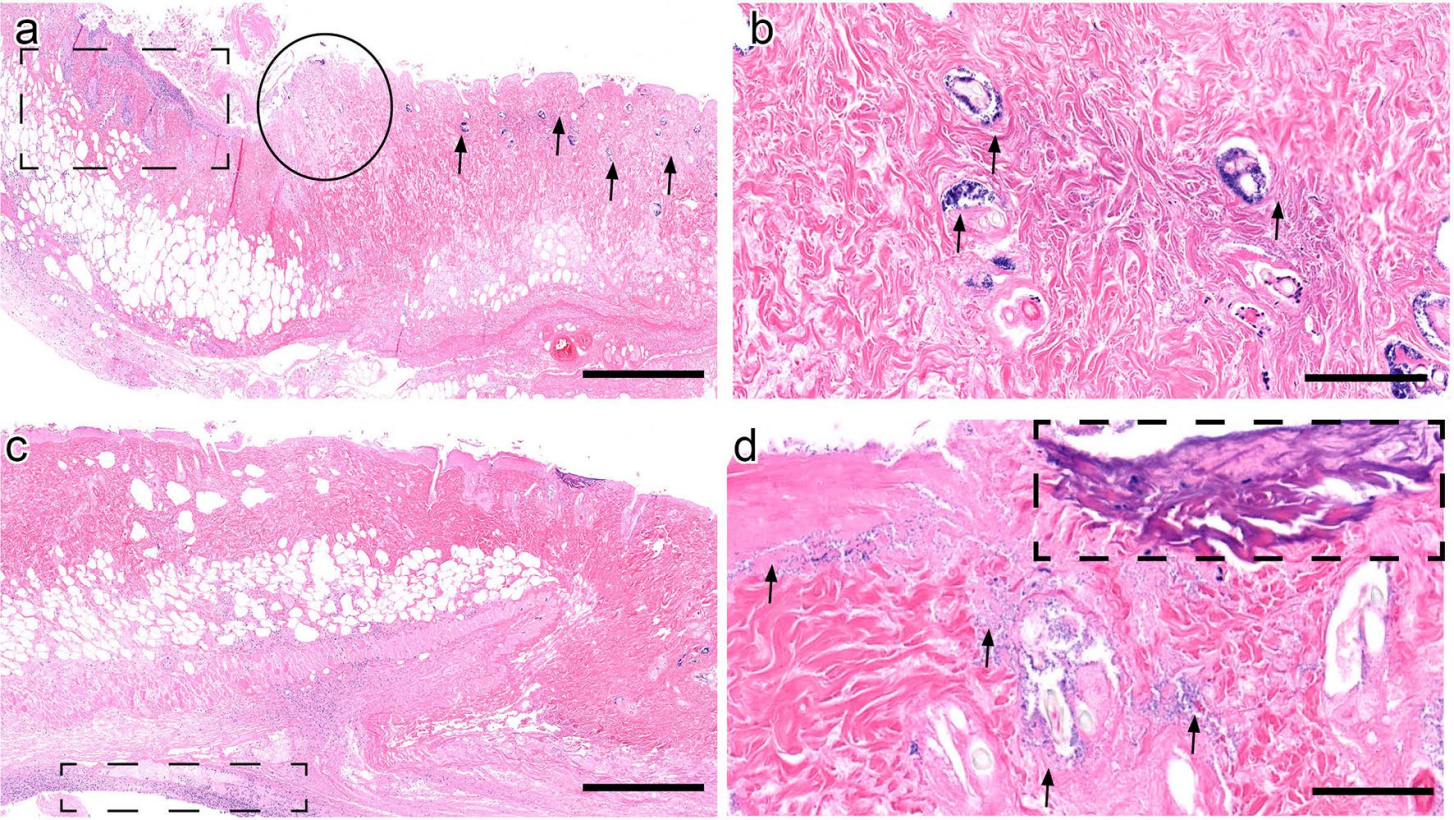
Histopathology of murine pressure ulcers infected with MRSA and treated with oral clindamycin or topical auranofin alone for four days. (**A**) Clindamycin-treated animals demonstrated the most pronounced wound healing with wound contrition (circle) and very few bacterial colonies present in the dermis (arrows). Inflammation was minimal and peripheral to the wound (dashed black box). Wounds demonstrated further resolution of inflammation and angiogenesis in the deep subcutis (solid black box). Scale bar represents 1 mm. (**B**) On closer view, bacteria are present in clusters within hair follicles (arrows) (image acquired at 2 × magnification). Scale bar represents 100 μm. (**C**) Auranofin-treated ulcers revealed a marked decrease in bacterial burden compared to systemic clindamycin treatment with mild inflammation deep in the subcutis (dashed box) (image acquired at 2 × magnification). Scale bar represents 1 mm. (**D**) Some foci of superficial mineralization (dashed box) were seen in auranofin-treated animals (image acquired at 20 × magnification). Scale bar represents 100 μm.

In order to further evaluate the impact of auranofin, mupirocin, and clindamycin treatment on wound healing in the diabetic mouse model, MRSA-infected wounds were evaluated using histopathology. Systemic clindamycin administration demonstrated few superficial bacteria, with coccobacilli confined to hair follicles in the superficial dermis and at the superficial ulcerated wound margin (Fig. 2A, arrows). Peripheral margins of wounds demonstrated contraction, indicating early healing (Fig. 2A, circle). Inflammation was confined to the superficial dermis (Fig. 2A, dashed box) with resolution in the deep subcutis, and evidence of early angiogenesis was present (Fig. 2A, solid box). On closer view, bacterial colonies formed clusters within hair follicles (Fig. 2B, arrows). Topical auranofin administration resulted in a marked decrease in bacterial burden, confined to the superficial ulcerated regions (Fig. 2C). However, compared to clindamycin treatment, peripheral inflammation deep in the subcutis persisted (Fig. 2C, dashed box), and no angiogenesis was seen. However, the bacterial burden was markedly decreased compared to clindamycin (Fig. 2D, arrows). Foci of mineralization were present at the wound surface (Fig. 2D, dashed box) in contrast to conventional treatment. Wound healing was advanced in both clindamycin and auranofin groups compared to vehicle controls.

**Figure 3:**
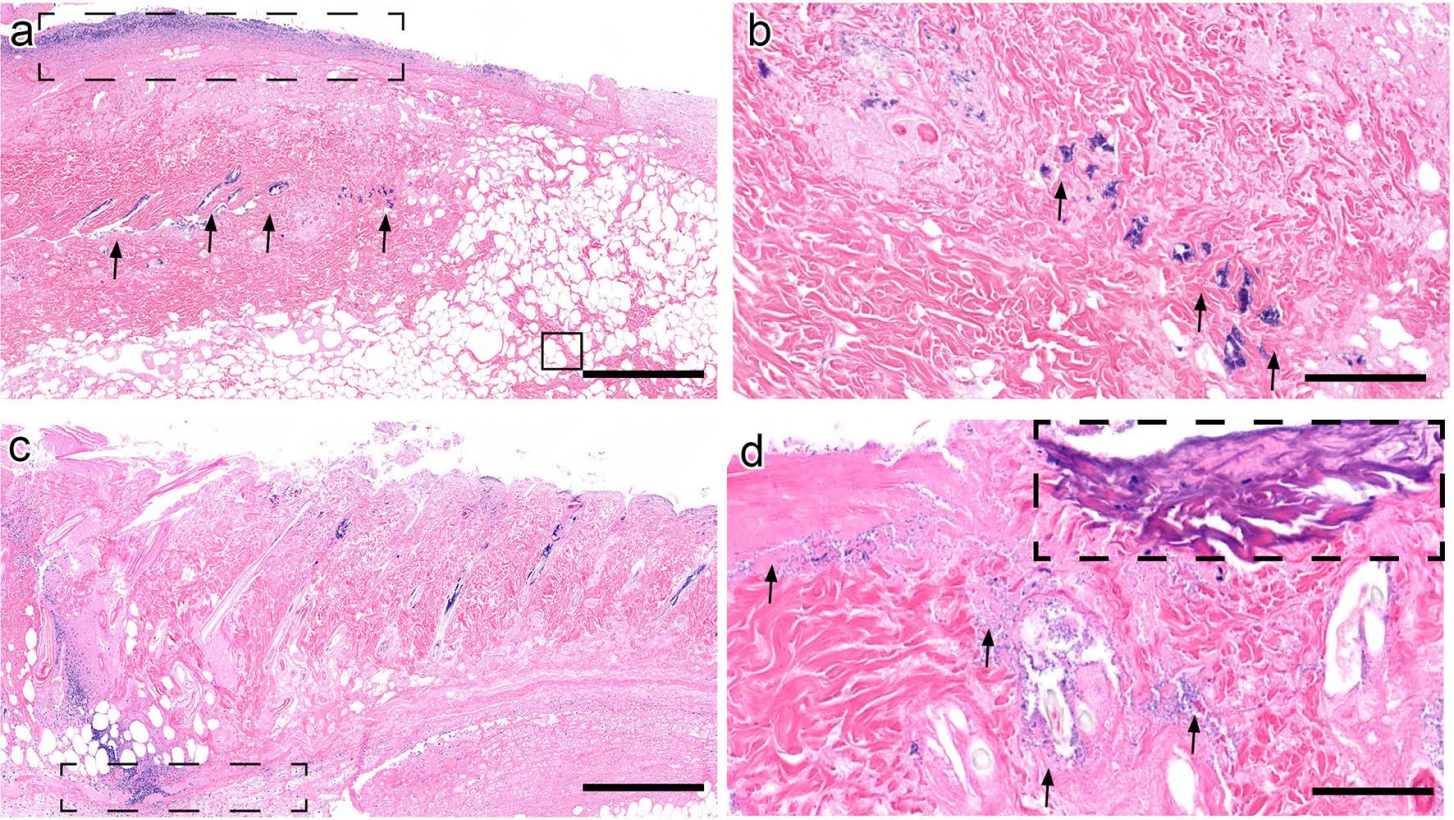
Histopathology of murine pressure ulcers infected with MRSA and given vehicle control or topical mupirocin treatment. **(A**) In vehicle control animals, pressure ulcers were severe, with necrosis extending into the deep dermis and subcutis. A myriad of superficial bacteria were present as a crust on the ulcerated lesion surface (dashed box). Bacterial colonies often extended into the deep dermis (arrows) (image acquired at 2 × magnification). Scale bar represents 1 mm. (**B**) A closer view showing bacterial colonies clustered within hair follicles and scattered in the dermis (arrows) (image acquired at 20 × magnification). Scale bar represents 100 μm. (**C**) Mupirocin-treated animals demonstrated less superficial mats of bacterial colonies compared to vehicle controls, with bacteria still present in hair shafts (arrows). A peripheral margin of wound contraction was seen in mupirocin groups (circle), which was absent in untreated controls. A peripheral front of inflammation was seen in mupirocin-treated animals (dashed box) compared to vehicle controls. Scale bar represents 1 mm. (**D**) On 20 × magnification, bacteria were fewer and confined to the superficial dermis or hair follicles (arrows).

In vehicle control animals, pressure ulcers were severe, extending full thickness into the deep dermis. Superficial bacteria were innumerable and presented as a crust on the ulcerated lesion surface (Fig. 3A, dashed box). Bacterial colonies extended into the deep dermis (Fig. 3A, arrows). Bacterial colonies formed numerous clusters within hair follicles and scattered in the dermis (Fig. 3B, arrows). Mupirocin-treated animals demonstrated less superficial mats of bacterial colonies compared to vehicle controls, with bacteria present in hair shafts (Fig. 3C, arrows). A peripheral margin of superficial contraction was seen in mupirocin groups (Fig. 3C, circle), indicating early wound healing, compared to untreated controls. A peripheral margin of inflammation was seen in mupirocin-treated animals (Fig. 3C, dashed box) compared to untreated controls. On higher magnification, bacteria were less numerous and confined to the superficial dermis or hair follicles (Fig. 3D, arrows).

### Auranofin is safe when applied topically to porcine skin

**Figure 4:**
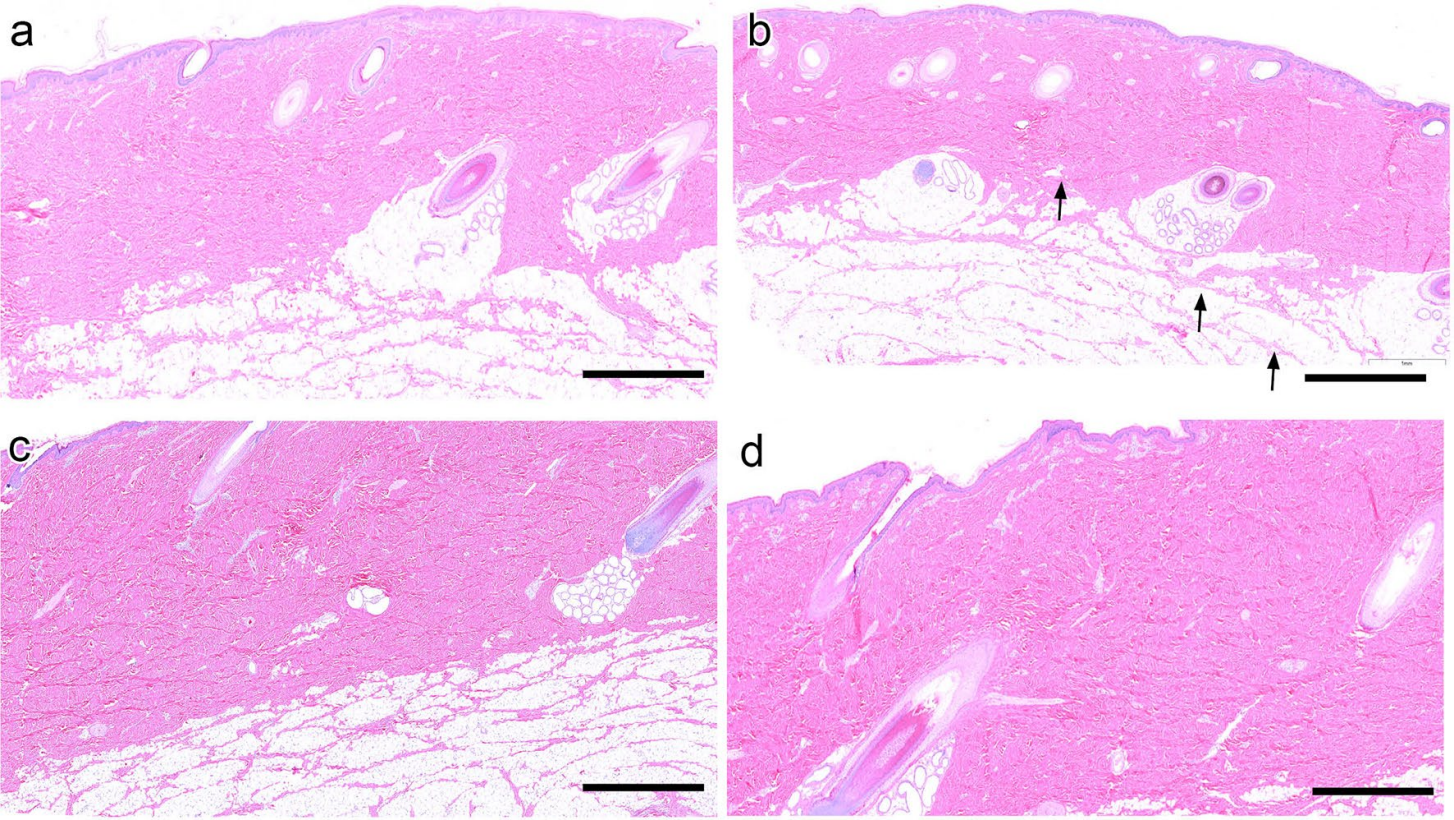
Histopathology of porcine skin tissues exposed to topical auranofin for 4, 7, or 14 days. Cutaneous sites exposed to topical auranofin were excised, formalin fixed, and evaluated using histopathology. No significant histologic lesions were seen in any drug applied topically, including toxicologic effects. (**A**) Vehicle control. (**B**) Pig 2, 2% auranofin for 4 days. (**C**) Pig 3, 1% auranofin for 14 days. (**D**) Pig 3, 3% auranofin for 14 days.

Conducting toxicological assessment of relevant tissues exposed to a new compound/drug candidate is a necessary step in pre-clinical studies. As this study proposed using auranofin as a topical agent to treat diabetic PUs infected with MRSA, evaluating auranofin for potential dermal irritation and toxicity was important. We chose to evaluate dermal toxicity of auranofin using pigs, as porcine skin is more similar anatomically to human skin compared to other animal species used for toxicology studies, including rodents, rabbits, and guinea pigs^21,22^. To conduct this assessment, topical auranofin (2%) was initially applied b.i.d to three different sites on the dorsum of one pig (pig 1) for four days, similar to the treatment plan used for the murine MRSA-infected PU study. Skin sites where auranofin was applied were monitored regularly for signs of erythema, edema, discoloration, or irritation. No signs of dermal irritation or toxicity were observed for skin sites exposed to auranofin (Fig. 4B). When treatment with topical auranofin (2%) was extended to seven days, once again no signs of erythema, edema, or irritation were observed on porcine skin (pig 2) where auranofin was applied. Finally, we evaluated auranofin at two different concentrations (1 and 3%), three skin sites per concentration, applied over 14 days to determine if there would be a concentration- or time-dependent effect observed with regards to dermal toxicity (pig 3). Auranofin was applied to skin sites for two weeks based upon guidelines from the International Working Group on the Diabetic Foot and the Infectious Diseases Society of America that recommend antibiotics be administered for one to two weeks for the treatment of diabetic foot infections, including mild-to-moderate infected PUs^8,23^. Once again, no significant lesions were observed on porcine skin where either 1% or 3% auranofin was applied topically for 14 days (Fig. 4C,D).

### Plasma gold concentration in pigs after repeated exposure to topical auranofin

**Figure 5:**
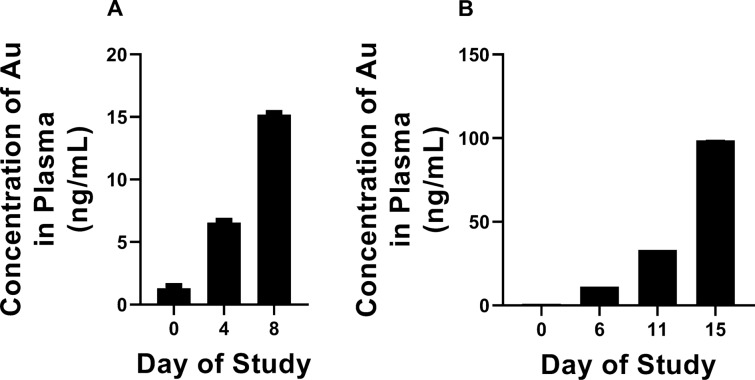
Serum gold concentration in pigs treated with topical auranofin for 7 or 14 days. Male castrate Landrace cross pigs were treated with topical auranofin for (**A**) 7 days (pig 2, 2% auranofin b.i.d.) or (**B**) 14 days (pig 3, 1% or 3% auranofin b.i.d.). Blood samples were obtained in metal-free tubes before exposing pigs to auranofin (day 0) and on days 4 or 8 (for pig 2) or on days 6, 11, and 15 (for pig 3) of the study. Samples were centrifuged and plasma was analyzed for total gold concentration (corresponding to auranofin) via ICP-MS.

Transdermal penetration of compounds/drugs intended to be applied topically can result in drugs accumulating in systemic circulation, which could result in undesirable side effects^24^. To investigate if auranofin would be able to permeate intact porcine skin exposed to the drug, blood samples were obtained from pig 2 (2% auranofin for seven days) and pig 3 (1 and 3% auranofin for 14 days) at several time points throughout the study. Auranofin undergoes rapid metabolism to the active gold form after systemic administration; thus we measured plasma gold concentration in pigs as a metric of auranofin accumulation, as reported in a recent Phase I clinical trial evaluating auranofin’s safety profile in humans^25^. In pig 2, there was minimal detection of gold in plasma samples obtained before treatment was initiated (1.314 ± 0.057 ng/mL) and after days 4 (6.565 ± 0.028 ng/mL) and 8 (15.199 ± 0.013 ng/mL) of the study (Fig. 5A). The gold concentration was higher in pig 3, which received topical auranofin for 14 days (Fig. 5B). Compared to before treatment (0.968 ± 0.018 ng/mL), plasma levels increased moderately on days 6 (11.339 ± 0.007 ng/mL), 11 (33.185 ± 0.010 ng/mL), and 15 (98.627 ± 0.004 ng/mL).

### Evaluation of markers for systemic toxicity in pigs exposed to topical auranofin

To confirm there were no adverse systemic side effects observed with topical administration of auranofin to porcine skin, blood samples were collected and submitted for a complete metabolic profile and complete blood count (Supplementary Table S1). In pig 2 treated with 2% auranofin for seven days, the levels of creatinine (ranged from 1.10 to 1.30 mg/dL), albumin (ranged from 2.8 to 3.2 g/dL), aspartate aminotransferase (ranged from 34 to 58 IU/L), alkaline phosphatase (ranged from 156 to 181 IU/L), and total bilirubin (0.20 mg/dL) were all within reference ranges indicating no signs of kidney, liver, or gallbladder dysfunction. Additionally, CO_2_ values (ranged from 30 to 34 mM) remained in close proximity to the baseline measurement indicating no imbalance in pH, oxygen, or carbon dioxide levels in the blood, which would be indicative of metabolic derangements. In the CBC, hematocrit (32.4–41.0%) and hemoglobin (10.2–12.5 g/dL) levels were within normal ranges indicating no evidence of anemia. Furthermore, white blood cell counts (16.2–19.4 K/µL) were neither diminished nor elevated indicating no signs of neutropenia or infection were present in pig 2 through the duration of the study.

For pig 3 treated with 1% or 3% topical auranofin for 14 days, no signs of kidney, liver, lung, or gallbladder dysfunction were observed from the complete metabolic profile data (Supplementary Table S1). Values for creatinine (ranged from 1.10 to 1.30 mg/dL), albumin (ranged from 2.8 to 3.2 g/dL), aspartate aminotransferase (ranged from 34 to 38 IU/L), alkaline phosphatase (ranged from 142 to 201 IU/L), total bilirubin (ranged from < 0.10 to 0.20 mg/dL), and CO_2_ (ranged from 31 to 34 mM) were all within reference ranges. Additionally, for pig 3, the hematocrit (ranged from 34.2 to 38.9%) and hemoglobin (ranged from 10.9 to 12.4 g/dL) values were within normal ranges indicating the absence of anemia. Furthermore, white blood cell counts (ranged from 13.9 to 17.8 K/ µL) were neither diminished nor elevated, compared to the initial baseline value, indicating the absence of neutropenia or infection during treatment. Overall, exposure to 1%, 2%, and 3% auranofin was safe when administered topically to pigs.

## Discussion

Pressure ulcers are a significant concern for individuals with diabetes and often develop in lower extremities. More than 15% of individuals with a diabetic foot ulcer (DFU) will suffer amputation of the affected limb resulting in over 100,000 amputations in the U.S. alone each year^5^. In addition to enhanced risk of amputation, infected DFUs result in increased healthcare costs and mortality^4,9,11^. Bacterial infection of a DFU hinders healing of ulcerated tissues which contributes to treatment failure, formation of chronic non-healing wounds, and ultimately amputation of infected limbs^8^.

Currently, only three antibiotics (ertapenem, linezolid, and piperacillin/tazobactam) are FDA-approved in the U.S. for treatment of diabetic foot infections^9^. However, these newer antibiotics are more expensive to use (thus increasing treatment costs) and have not been shown to be superior to older antibiotics, such as vancomycin, in treatment of MRSA-infected diabetic foot infections^9^. Additionally, ertapenem is ineffective against MRSA^26^. Furthermore, ertapenem and piperacillin/tazobactam are associated with adverse skin rashes/reactions with prolonged use^27–29^. Thus, current guidelines recommend treating mild-to-moderate MRSA-infected pressure ulcers with an oral/parenteral antibiotic such as clindamycin or vancomycin for 10–14 days^8,23^. In severely infected DFUs, antibiotic treatment may need to be extended for up to three weeks^8^. However, prolonged use of systemic antibiotics, including clindamycin and vancomycin, is associated with toxicity (gastrointestinal or nephrotoxicity) and dysbiosis^30,31^. Previous guidelines included the use of topical antibiotics, such as mupirocin and retapamulin, for the treatment of mild infected DFUs^9^. Use of topical antibiotics is thought to be beneficial in enhancing concentration of drug at the site of infection while avoiding systemic toxicities associated with oral/parenteral antibiotics^9,32^. However, concerns about a lack of superiority of topical antibiotics relative to oral/parenteral antibiotics and promoting resistance to antibiotics that can be administered both topically and systemically resulted in topical antibiotics being removed from the most recent treatment guidelines for infected diabetic pressure ulcers^8^. Though antibiotics may be capable of reducing bacterial burden in PUs, they do not typically aid with healing of wounded tissues. Thus, there is a clear need for finding novel therapeutics capable of rapidly eliminating the burden of bacteria in mild-to-moderate infected diabetic pressure ulcers and promoting wound healing.

In the pursuit of novel agents to treat infected diabetic pressure ulcers, we identified auranofin as a promising candidate. Auranofin was originally approved as a second-line oral drug for treatment of rheumatoid arthritis. More recently, auranofin has received interest in being repurposed as an antibacterial agent^33–36^. We suspected auranofin would be a promising topical agent to treat diabetic pressure ulcers infected with MRSA as auranofin possesses potent antibacterial and antibiofilm activity in vitro and in vivo; additionally, auranofin reduced expression of pro-inflammatory cytokines (TNF-α, IL-1β, and IL-6) associated with impaired wound healing in uncomplicated cutaneous abscesses in mice^16,20^. Moreover, MRSA was found to be unable to develop rapid resistance to auranofin in vitro in single-step and multi-step resistance selection assays^15,37^. Furthermore, we recently demonstrated that auranofin was superior to both oral clindamycin and topical mupirocin in rapidly eradicating MRSA from pressure ulcers in obese mice^37^. This provided the impetus to evaluate auranofin for the treatment of MRSA-infected PUs in diabetic mice.

As 95% of individuals with diabetes exhibit Type 2 diabetes, we reviewed different mice models that would be suitable for the MRSA-infected pressure ulcer study^38^. One of the most widely used models for Type 2 diabetes research are B6.BKS(D)-*Lepr*^*db*^/J (or *db*/*db*^*−/−*^*)* mice. However, concerns with *db*/*db*^*−/−*^ mice include diminishing blood glucose concentration after four weeks of age and difficulty in maintaining hyperglycemia, a crucial trait of Type 2 diabetes^38^. Furthermore, *db*/*db*^*−/−*^ mice have difficulty maintaining body temperature and are susceptible to hypothermia, particularly after infection with bacteria^39,40^. We observed this in a pilot study of MRSA-infected PUs in *db*/*db*^*−/−*^ mice. Once PUs formed and were subsequently infected, mice often developed hypothermia resulting in death or euthanasia within 48–72 h after treatment was initiated. This led us to investigate alternative mouse models for Type 2 diabetes. One of the newer models in this regard are male TALLYHO/JngJ mice, which exhibit hyperinsulinemia and hyperglycemia between 10 to 14 weeks after birth^38^. The diabetic phenotype is stable in mice, and they develop mild-to-moderate obesity, as is representative of most human patients clinically^38^. Additionally, TALLYHO/JngJ mice were found to be more resilient than *db/db*^*−/−*^ mice, did not develop hypothermia, and were able to withstand formation of MRSA-infected PUs. Yet, it is important to note that the diabetic phenotype will not occur in all male TALLYHO/JngJ mice. Thus, measuring blood glucose levels is an important step to confirm hyperglycemia is present before initiating an experiment. Utilizing our mouse model, we determined that topical auranofin (2%) successfully eradicated MRSA within infected PUs of diabetic TALLYHO/JngJ mice after only four days of treatment. This was superior to both mupirocin and clindamycin and matched the result obtained with auranofin treatment of MRSA-infected PUs in obese mice^37^.

After confirming that auranofin rapidly eradicates MRSA in infected diabetic PUs in TALLYHO/JngJ mice, we next investigated the impact of treatment on biomarkers related to wound healing. In mice, skin wounds heal via contraction whereas in humans, skin wounds heal via formation of granulation tissue and re-epithelization^41^. This is due in part to a dense band of dorsal muscle present in mice^41,42^. Thus, measuring changes in wound diameter in mice over time may not provide an accurate assessment for the impact of a potential compound/drug on wound healing. Instead, measuring expression of specific biomarkers in skin wounds in response to treatment and conducting histopathology of the affected tissue provides a more complete picture of wound healing. Wound healing in mice and humans is a dynamic process that involves four stages; hemostasis, inflammation, proliferation, and tissue remodeling^43^. During the inflammation and proliferation stages, there is a delicate balance of cytokines and growth factors that are released to ensure the wound heals normally. Several cytokines (including IL-1, IL-6, TNF-α) and growth factors (including IGF and VEGF) play a critical role in resolution of inflammation, stimulation of new blood vessel formation, and synthesis of collagen that permit formation of granulation tissue^43^. However, the presence of bacteria in the wound bed interferes with granulation tissue formation^44,45^. Additionally, metabolic dysfunction associated with diabetes negatively impacts the synthesis of key proteins, collagen, and fibroblasts that results in wounds stalling in the inflammatory stage and not transitioning to re-epithelization^10^. This is due to a decreased production of growth factors (including IGF and VEGF), increased expression of pro-inflammatory cytokines (including IL-1β and IL-17), and elevated levels of matrix metalloproteinases (including MMP-2 and MMP-9)^43,45–47^. Collectively, this negatively impacts angiogenesis, deposition of collagen, and formation of granulation tissue, which results in non-healing PUs and chronic wounds^43^. To address these issues, researchers have investigated the use of exogenous growth factors and inflammatory mediators to enhance healing of PUs. To date, only one agent, becaplermin (human platelet-derived growth factor), has received FDA-approval for the treatment of diabetic pressure ulcers. However, becaplermin has not been shown to consistently reduce the time for diabetic PUs to heal clinically and is also expensive^9^. Additionally, the exogenous growth factors and inflammatory mediators studied are not able to reduce bacterial burden in infected diabetic PUs. Identifying agents capable of eliminating bacterial burden in infected PUs and enhancing wound healing would be ideal. In this regard, we evaluated the effect of auranofin treatment on MRSA-infected PUs in diabetic mice. Auranofin treatment resulted in decreased expression of pro-inflammatory cytokines (including IL-1β and IL-17) and matrix metalloproteinases (MMP-2 and MMP-9), relative to mice receiving the vehicle alone. Additionally, treatment of infected PUs with auranofin resulted in increased expression of several growth factors including IGF, VEGF, and GM-CSF. Furthermore, histopathology evaluation of PUs after only four days of treatment revealed that auranofin appeared to enhance healing of PUs compared to the vehicle alone and topical mupirocin.

The final step in our study was to complete a dermal toxicity assessment for auranofin. Auranofin has been investigated as an oral drug in multiple animal models and in human clinical trials. A recently completed phase I clinical trial conducted in humans determined oral auranofin to be a safe, anti-infective therapeutic when used for a short term (e.g. 6 mg daily for 7 days), with no adverse side effects observed^25^. Extending treatment for a longer course, if needed, would be expected to be safe as a 28-day course of 9–12 mg oral auranofin was not associated with adverse side effects for the treatment of chronic lymphocytic leukemia in a clinical trial (Clinical Trials registration number: NCT01419691). Furthermore, multiple studies investigating auranofin as a treatment for rheumatoid arthritis found no serious adverse side effects in patients receiving oral auranofin for 2–4 years^48,49^. However, to date, no studies have been conducted to evaluate dermal toxicity for auranofin. This is an important point to investigate in order to repurpose auranofin as a topical agent to treat MRSA-infected diabetic pressure ulcers.

Multiple animal models have been utilized to investigate dermal toxicity of xenobiotics, including rodents and rabbits. However, pigs are rapidly emerging as a preferred model for conducting dermal irritation and toxicity studies, as porcine skin more closely resembles human skin compared to other animal models^21,22^. Porcine skin and human skin have an epidermis that resembles a similar thickness and number of cell layers while also possessing both Langerhans cells and melanocytes^21^. Additionally, the main barrier to transdermal penetration of drugs, the stratum corneum, is similar structurally in both porcine and human skin making pigs a good model for evaluating drug candidates^21^. Furthermore, the dermis in both human and porcine skin contains blood vessels, elastic fibers, and collagen^21^. Pigs also possess several advantages over other animal models as it pertains to conducting dermal toxicity assessments of drugs. First, rodent and rabbit skin tends to be thinner than porcine skin; as a result, rodent and rabbit models tend to overestimate dermal irritation and toxicity compared to human skin^21^. Secondly, the size of pigs allows for one to evaluate multiple compounds or different concentrations of one test agent on a single pig, which reduces interindividual variation and the number of animals needed to complete a study^21^. To avoid cross-contamination, it is recommended to mark a maximum of six sites (5 cm × 5 cm or 2 inches × 2 inches per site) on a single pig, as we did in our study ^21^. Both farm pigs (such as those used in our study) and mini-pigs are acceptable models for use in dermal irritation and toxicity studies^21,22^.

Initially, we evaluated topical auranofin (2%) applied twice daily for four days to different sites on porcine skin to mimic the PU study conducted in diabetic mice. No dermal irritation or toxicity was observed with auranofin treatment. Thus, we repeated the study and extended treatment to seven days. Once again, no signs of erythema or edema were observed at the site of application. We then evaluated toxicity of auranofin at lower (1%) and higher (3%) concentrations over the course of 14 days (similar to the current treatment guidelines for antibiotics prescribed for mild-to-moderate infected diabetic PUs). Previously, we found that 1% auranofin effectively eradicated MRSA in infected PUs of obese mice^37^. The higher dose (3%) was chosen to determine if dose-dependent toxicity would be observed. At both the lower and higher doses, no adverse effects were observed on the skin sites of the treated pig, indicating auranofin is a safe topical agent. Furthermore, no systemic side effects were observed in pigs treated with 2% auranofin topically for seven days (pig 2) or 1% or 3% auranofin for 14 days (pig 3). The maximum plasma gold concentration was 15.199 ± 0.013 ng/mL for pig 2 and was 98.627 ± 0.004 ng/mL for pig 3. This concentration is still far lower than the maximum plasma gold concentration of 0.312 µg/mL determined in a clinical trial in humans where 6 mg auranofin was given orally for seven days^25^. No adverse side effects were observed in human patients exposed to 0.312 µg/mL gold. This suggests that topical auranofin (up to 3%) applied twice daily over 14 days would not be expected to result in serious local or systemic side effects. This was further confirmed in a complete metabolic profile and complete blood count assessment where levels of creatinine, albumin, aspartate aminotransferase, alkaline phosphatase, total bilirubin, and CO_2_ were all within reference ranges for both pigs 2 and 3. Additionally, the hematocrit, hemoglobin, and white blood cell counts were neither diminished nor elevated, which indicated no signs of anemia, neutropenia or infection were present in pigs 2 and 3 through the duration of the study.

As noted above, recent guidelines for the treatment of mild-to-moderate infected PUs removed the use of topical antibiotics due to lack of superiority compared to systemic antibiotics and concerns about promoting antibacterial resistance. However, auranofin is not currently approved for use as a systemic antibiotic and has shown minimal likelihood in vitro of bacterial resistance forming rapidly. Furthermore, in this study, auranofin demonstrated superiority against currently approved antibiotics in eradicating MRSA in infected PUs in diabetic mice and was safe when applied topically to porcine skin. This suggests auranofin warrants further investigation as a novel topical treatment option for mild-to-moderate MRSA-infected diabetic pressure ulcers.

## Methods

### Bacterial strain and reagents

MRSA NRS384 (USA300) was acquired from the Biodefense and Emerging Infections Research Resources Repository (BEI Resources, Manassas, VA, USA). Auranofin, clindamycin, and mupirocin were purchased from commercial vendors. Mannitol salt agar, phosphate-buffered saline (PBS), petroleum jelly, rare earth magnets, betadine, buprenorphine, euthasol, formalin, Tegaderm, Uro-bond IV, metal-free centrifuge tubes, EDTA tubes, and serum tubes were also purchased from commercial vendors.

### Diabetic pressure ulcer mouse model

The reporting in the manuscript of all animal studies conducted follows the recommendations in the ARRIVE guidelines. The mice study was reviewed and approved by the Purdue Animal Care and Use Committee and conducted in strict accordance with the National Institutes of Health Guide for the Care and Use of Laboratory Animals. Twelve-week old male TALLYHO/JngJ mice (Jackson Laboratory, Bar Harbor, ME), weighing between 33.2 to 39.2 g, were used for this experiment. Mice were fed a high fat diet and blood samples were obtained to determine the blood glucose concentration using a glucose home monitoring kit (concentrations ≥ 130 mg/dL for mice in a fasting state were diabetic, per the manufacturer’s guidelines). Pressure ulcers were created using rare earth magnets and infected with 20 µL of 9.0 × 10^8^ CFU/mL MRSA NRSA384, using a previously published protocol^37^. This method resulted in full-thickness wounds with erythema around the edge of the ulcer. These MRSA-infected ulcers were treated topically b.i.d with the vehicle alone (petroleum jelly, negative control), 2% auranofin, or 2% mupirocin (positive control) for four days. One group of mice received oral clindamycin (30 mg/kg) q.d. for four days. Each group (negative control, auranofin, mupirocin, and clindamycin) consisted of five mice. PUs were covered with Tegaderm sealed with Uro-bond IV to minimize grooming of the affected area and removal of the test agent. The doses and duration of treatment were based upon a previous study conducted in female obese TALLYHO/JngJ mice^37^. Twelve hours after the last dose was administered, all mice were euthanized via CO_2_ asphyxiation. Infected PUs were harvested aseptically, the right PU was homogenized in sterile PBS (Omni Tissue Homogenizer TH115, Omni International, Kennesaw, GA, USA), serially diluted in PBS, and aliquots from each dilution were plated on mannitol salt agar plates. Plates were incubated for at least 20–24 h at 37 °C before MRSA colonies were enumerated. As no growth was observed on plates for auranofin samples after 24 h, these plates were incubated for up to 48 h to confirm the absence of colonies. Additionally, 1 mL of skin homogenate from auranofin-treated ulcers was spread onto five different mannitol salt agar plates and incubated for 48 h at 37 °C as a secondary confirmation for the absence of colonies. Data are presented as log_10_ (MRSA CFU) determined from the infected pressure ulcer from each mouse. Cytokines analysis and histopathology evaluation of infected PUs are presented in the Supporting Information file.

### Cutaneous toxicity evaluation of auranofin on porcine skin

The porcine dermal toxicity study was reviewed and approved by the Purdue Animal Care and Use Committee. Seven-month old male castrate Landrace cross pigs (Animal Sciences Research and Education Center Swine Unit, Purdue University, West Lafayette, IN) were housed in individual holding pens through the duration of the study with access to food and water. The dorsum was shaved and six sites (2-inch by 2-inch) were marked on each pig. The first and second pigs were treated with either topical auranofin (2%) or the vehicle alone (petroleum jelly), three sites per treatment, b.i.d. (approximately seven hours between doses) for either four (pig 1) or seven (pig 2) days. A third pig (pig 3) was treated with topical auranofin, either 1% or 3% applied to three different sites per concentration, b.i.d. for 14 days. The treated skin sites were covered with Tegaderm between doses to ensure pigs did not groom or remove the test agent. Treated skin tissues were monitored for signs of erythema and edema at the application sites. Twelve hours after the final dose was administered, pigs were humanely euthanized with euthasol (sodium pentobarbital 390 mg/mL at 1 mL/10 lb of body weight). The skin tissues where drug or vehicle were applied were subsequently harvested aseptically for analysis. Histopathology evaluation of porcine skin and determination of plasma gold concentration and serum biochemistry are presented in the Supporting Information file.

### Statistical analyses

The bacterial CFU counts in the MRSA-infected pressure ulcers in diabetic mice were analyzed via the Kruskal–Wallis test with Dunn’s multiple comparisons test (*P* < 0.05) using GraphPad Prism8 (La Jolla, CA).

## Supplementary Information

Supplementary Information.
