# Supplementary material for: Investigating auranofin for the treatment of infected diabetic pressure ulcers in mice and dermal toxicity in pigs
Source: Sci Rep. 2021 May 25;11:10935. doi: 10.1038/s41598-021-90360-x (PMC8149385; doi:10.1038/s41598-021-90360-x)
Supplement: Supplementary file 1 — Supplementary Information. [file 41598_2021_90360_MOESM1_ESM.docx]

**Investigating Auranofin for the Treatment of Infected Diabetic Pressure Ulcers in Mice and Dermal Toxicity in Pigs**

Haroon Mohammad^1^, Nader S. Abutaleb^1^, Alexandra M. Dieterly^1^, L. Tiffany Lyle^1,2^, and Mohamed N. Seleem^1,3*^

^1^ Department of Comparative Pathobiology, College of Veterinary Medicine, Purdue University, 625 Harrison St., West Lafayette, IN 47907, USA

^2^ Center for Comparative Translational Research, Purdue University, 625 Harrison St., West Lafayette, IN, 47907, USA

^3^ Department of Biomedical Sciences and Pathobiology, Virginia-Maryland College of Veterinary Medicine, Virginia Polytechnic Institute and State University, Blacksburg, VA 24061

***Corresponding Author:**

Mohamed N. Seleem

Center for One Health Research

Virginia-Maryland College of Veterinary Medicine

1410 Prices Fork Rd, Blacksburg, VA 24061, USA

Phone: 540-231-2702

Email: seleem@vt.edu

**Short title**: Auranofin treatment of diabetic ulcers

**Supplementary Materials and Methods**

**Cytokines analysis of infected pressure ulcers in diabetic mice**

Skin homogenates obtained from the right PU of each mouse were centrifuged (10,000 rpm for 10 minutes) and the supernatant was transferred to a separate tube. For each treatment group, aliquots (0.1 mL) from samples from all five mice were pooled together. The total protein content for each sample was measured via the bicinchoninic acid assay, standardized, and expression of cytokines and growth factors was subsequently determined via the Quantibody Mouse Cytokine Array 4000 kit (RayBiotech Life, Norcross, GA). The fold change in cytokines expression and growth factors between each treatment group (auranofin, mupirocin, or clindamycin) relative to the negative control was calculated and presented as a heat map using GraphPad Prism8 (La Jolla, CA).

**Histopathologic evaluation of MRSA-infected pressure ulcers in diabetic mice**

Pressure ulcers (left wound) from infected mice in all four groups of diabetic mice (vehicle, auranofin, clindamycin, and mupirocin) were collected after euthanasia and evaluated histologically. Sections of affected skin were removed en bloc and placed in room temperature 10% neutral-buffered formalin for 24 hours. Tissues were processed over 10 hours using a Sakura Tissue-Tek VIP6 tissue processor. Tissues were processed sequentially in 70%, 80%, 95%, and 100% ethanol, followed by xylene and paraffin, and were embedded in Surgipath Paraplast Plus (Leica Biosystems, Wetzlar, Germany). Tissue sections, 4-µm thickness, were placed on charged slides, stained with hematoxylin and eosin, and cover- slipped using a Leica ST5010-CV5030 integrated workstation.

**Histopathology evaluation of porcine skin exposed to auranofin for evidence of dermal toxicity**

Porcine skin tissues were harvested immediately after pigs were euthanized. Sections of affected skin and underling subcutis and skeletal muscle were removed en bloc and placed in room temperature 10% neutral-buffered formalin for 24 hours. Tissues were processed as described previously. Skin samples were evaluated microscopically in parallel to vehicle controls (petroleum jelly).

**Determination of plasma gold concentration from pigs exposed to auranofin**

Blood samples were collected before treatment was initiated and after treatment with topical auranofin on days 4 and 8 (12 hours after the final dose was administered) for pig 2 or on days 6, 11, and 15 (12 hours after the final dose was administered) for pig 3. Samples were centrifuged (4000 × *g* for five minutes) and plasma was transferred to metal-free centrifuge tubes (Labcon, Petaluma, CA). Plasma samples were digested in 70% trace metal grade nitric acid and maintained at 70°C in a water bath incubator overnight. Thereafter, 30% trace metal grade hydrogen peroxide was added to each sample and kept at 70°C for two hours. Samples were then diluted with trace metal grade water. The digested samples were assayed by ICP-MS NexION 300D (PerkinElmer, Inc., Shelton, CT, USA) with a resolution equal to 0.9 amu at 10% peak height from 6-253 amu and 1.0 amu at 5% peak height from 6-253 amu, with corrections for isobaric interferences. ICP-MS was operated in standard mode. Bi was used as an internal standard. Calibration curves were constructed using a zero-point standard and a five-point calibration series. The R-squared value for the response function was greater than 0.999. Five replicates were analyzed per sample.

**Evaluation of markers for systemic toxicity for pigs exposed to auranofin**

Blood samples were collected before treatment and at specific time points after treatment with topical auranofin on days 4 and 8 (12 hours after the final dose was administered) for pig 2 or on days 5, 11 and 15 (12 hours after the final dose was administered) for pig 3. Blood was collected in EDTA tubes for complete blood count (CBC) determination and serum blood collection tubes for the Chem 12 panel. Samples were submitted to the Purdue Veterinary Clinical Pathology Laboratory to obtain a CBC and Chem 12 profile for relevant metabolic markers.

**Supplementary Table S1: Serum biochemistry and complete blood count values for pig 2 (topical auranofin (2%) for seven days) and pig 3 (topical auranofin (1% or 3%) for 14 days)**. Reference ranges for biomarkers measured are provided, where available.

|  | **PIG 2** | | | **PIG 3** | | | | **REFERENCE RANGE** |
| --- | --- | --- | --- | --- | --- | --- | --- | --- |
|  | **Pre-treatment** | **Day 4** | **Day 8** | **Pre-treatment** | **Day 5** | **Day 11** | **Day 15** |  |
| GLU (mg/dL) | 110 | 88 | 104 | 76 | 87 | 103 | 87 | 85 – 160 |
| BUN  (mg/dL) | <2 | 6 | 6 | 10 | 4 | 4 | 3 | 6 – 30 |
| CREA (mg/dL) | 1.30 | 1.10 | 1.10 | 1.10 | 1.20 | 1.10 | 1.30 | 0.50 – 2.10 |
| PHOS (mg/dL) | 10.0 | 9.7 | 10.0 | 6.7 | 8.0 | 8.2 | 8.8 | 3.6 – 9.2 |
| Ca (mg/dL) | 10.1 | 9.8 | 9.8 | 9.2 | 9.8 | 10.0 | 10.2 | 5.0 -11.4 |
| Na (mM) | 138 | 138 | 135 | 140 | 136 | 137 | 137 | 142 – 149 |
| K  (mM) | 3.9 | 4.6 | 3.9 | 3.5 | 4.0 | 4.8 | 3.6 | 2.1 – 7.1 |
| CL  (mM) | 98 | 99 | 98 | 106 | 99 | 102 | 98 | 100 – 109 |
| CO_2_ (mM) | 30 | 34 | 31 | 33 | 34 | 31 | 34 | N.D. |
| AGAP (mM) | 13.9 | 9.6 | 9.9 | 4.5 | 7.0 | 8.8 | 8.6 | N.D. |
| TP (g/dL) | 5.6 | 5.1 | 5.1 | 4.7 | 5.0 | 5.2 | 5.5 | 6.0 – 8.0 |
| ALB (g/dL) | 3.1 | 2.8 | 2.8 | 2.8 | 2.8 | 3.0 | 3.2 | 1.8 – 3.3 |
| GLOB (g/dL) | 2.5 | 2.3 | 2.3 | 1.9 | 2.2 | 2.2 | 2.3 | N.D. |
| A/G | 1.2 | 1.2 | 1.2 | 1.5 | 1.3 | 1.4 | 1.4 | 0.4 – 0.5 |
| AST (IU/L) | 58 | 49 | 43 | 34 | 34 | 38 | 35 | 16 – 65 |
| ALKP (IU/L) | 163 | 181 | 156 | 142 | 159 | 188 | 201 | 92 – 294 |
| GGT (IU/L) | 37 | 35 | 35 | 47 | 51 | 49 | 57 | 16 – 30 |
| TBIL (mg/dL) | 0.20 | 0.20 | 0.20 | <0.10 | 0.20 | 0.20 | 0.20 | 0.00 – 0.30 |
| Mg (mg/dL) | 2.0 | 1.8 | 1.7 | 1.4 | 1.8 | 1.7 | 1.7 | N.D. |
| CK (IU/L) | 2405 | 317 | 1378 | 147 | 229 | 542 | 912 | 50 – 3531 |
| **COMPLETE BLOOD COUNT (CBC)** | | | | | | | | |
| RBC (M/µL) | 7.84 | 6.54 | 6.38 | 6.72 | 6.76 | 6.41 | 7.22 | N.D. |
| HCT (%) | 41.0 | 33.5 | 32.4 | 36.6 | 35.8 | 34.2 | 38.9 | N.D. |
| HGB (g/dL) | 12.5 | 10.6 | 10.2 | 11.4 | 11.6 | 10.9 | 12.4 | N.D. |
| MCV (fL) | 52.3 | 51.3 | 50.7 | 54.4 | 52.9 | 53.4 | 53.8 | N.D. |
| MCHC (g/dL) | 30.4 | 31.5 | 31.4 | 31.2 | 32.4 | 31.8 | 31.9 | N.D. |
| RDW (%) | 15.5 | 16.1 | 16.6 | 15.6 | 15.7 | 15.6 | 16.1 | N.D. |
| WBC (K/µL) | 18.4 | 19.4 | 16.2 | 15.5 | 17.8 | 15.4 | 13.9 | N.D. |
| SEG  (K/µL) | 6.6 | 6.8 | 6.8 | 3.1 | 6.1 | 4.2 | 2.1 | N.D. |
| LYMPH (K/µL) | 10.8 | 11.5 | 8.9 | 11.0 | 11.1 | 9.8 | 11.7 | N.D. |
| MONO (K/µL) | 0.18 | 0.78 | 0.32 | 0.62 | 0.36 | 0.77 | 0.14 | N.D. |
| EOS (K/µL) | 0.37 | N.D. | 0.16 | 0.78 | 0.18 | 0.62 | N.D. | N.D. |

Abbreviations: GLU = glucose; BUN = blood urea nitrogen; CREA = creatinine; PHOS = phosphorous; Ca = calcium; Na = sodium; K = potassium; CL = chloride; CO_2_ = carbon dioxide; AGAP = anion gap; TP = total protein; ALB = albumin; GLOB = globulin; A/G = albumin to globulin ratio; AST = aspartate aminotransferase; ALKP = alkaline phosphatase; GGT = Gamma-glutamyl transferase; TBIL = total bilirubin; Mg = magnesium; CK = creatine kinase: RBC = red blood cell count; HCT = hematocrit; HGB = hemoglobin; MCV = mean corpuscular volume; MCHC = mean corpuscular hemoglobin concentration; RDW = red cell distribution width; WBC = white blood cell count; SEG = segmented neutrophils; LYMPH = lymphocyte count; MONO = monocyte count; EOS = eosinophil count; N.D. = not determined.
